# Cost-effectiveness of a mailed educational reminder to increase colorectal cancer screening

**DOI:** 10.1186/1471-230X-11-93

**Published:** 2011-08-25

**Authors:** Jeffrey K Lee, Erik J Groessl, Theodore G Ganiats, Samuel B Ho

**Affiliations:** 1 Department of Medicine, VA San Diego Healthcare System and University of California, San Diego, 3350 La Jolla Village Drive, San Diego, California 92161, USA; 2 Research Service, VA San Diego Healthcare System, 3350 La Jolla Village Drive, San Diego, California 92161, USA; 3 Department of Family and Preventive Medicine, University of California, San Diego; 9500 Gilman Drive, La Jolla, California 92093, USA

**Keywords:** Cost-effectiveness, Reminder, Colorectal Cancer, Screening, FOBT

## Abstract

**Background:**

Colorectal cancer (CRC) screening rates are low in many areas and cost-effective interventions to promote CRC screening are needed. Recently in a randomized controlled trial, a mailed educational reminder increased CRC screening rates by 16.2% among U.S. Veterans. The aim of our study was to assess the costs and cost-effectiveness of a mailed educational reminder on fecal occult blood test (FOBT) adherence.

**Methods:**

In a blinded, randomized, controlled trial, 769 patients were randomly assigned to the usual care group (FOBT alone, n = 382) or the intervention group (FOBT plus a mailed reminder, n = 387). Ten days after picking up the FOBT cards, a 1-page reminder with information related to CRC screening was mailed to the intervention group. Primary outcome was number of returned FOBT cards after 6 months. The costs and incremental cost-effectiveness ratio (ICER) of the intervention were assessed and calculated respectively. Sensitivity analyses were based on varying costs of labor and supplies.

**Results:**

At 6 months after card distribution, 64.6% patients in the intervention group returned FOBT cards compared with 48.4% in the control group (P < 0.001). The total cost of the intervention was $962 or $2.49 per patient, and the ICER was $15 per additional person screened for CRC. Sensitivity analysis based on a 10% cost variation was $13.50 to $16.50 per additional patient screened for CRC.

**Conclusions:**

A simple mailed educational reminder increases FOBT card return rate at a cost many health care systems can afford. Compared to other patient-directed interventions (telephone, letters from physicians, mailed reminders) for CRC screening, our intervention was more effective and cost-effective.

## Background

Colorectal cancer (CRC) is one of the leading causes of cancer-related deaths in the United States and a common cause of morbidity and mortality worldwide [1]. In 2008, about 148,000 new cases of CRC will be diagnosed, and about 50,000 people will die from this disease [2]. Several randomized controlled trials have shown evidence for the effectiveness of fecal occult blood testing (FOBT) in reducing CRC mortality by using samples from three successive stools [3,4]. In one randomized trial, annual FOBT followed by colonoscopy in those with a positive test reduced colorectal cancer mortality by 33% [5]. The impact of FOBT screening on CRC incidence and mortality is likely due to the early detection and removal of precancerous adenomatous polyps and earlier stage cancers.

National efforts have been made to increase awareness of CRC screening over the past several years. Recently, the United States Preventive Services Task Force (USPSTF) and National Comprehensive Cancer Network (NCCN) recommend all individuals aged 50 to 75 years, who are at average risk for CRC to use one of the following methods for CRC screening: an annual high sensitivity FOBT, a flexible sigmoidoscopy every 5 years, or a colonoscopy every 10 years [6,7]. Despite these recommendations and guidelines, CRC screening rates remain low. As of 2006, almost 50% of adults age 50 years or older were not up-to-date with CRC screening [2]. Numerous studies have shown that patient compliance in CRC screening programs and FOBT card return rates are suboptimal [8-11]. As a result, interest in cost-effective patient-directed interventions to promote CRC screening remains keen.

Recently, we reported that a mailed educational reminder increased FOBT card return rate for CRC screening by 16.2% (P < 0.001) at a large Veteran Affairs (VA) medical center [12]. However, it is not known if our intervention was cost-effective, particularly in the context of other similar cancer screening promotion programs. Given the highly constrained resources for health promotion, it is essential for decision makers to require information on the costs and cost-effectiveness of the interventions. Although there have been several studies on the economics of cervical cancer and mammography screening promotion [13,14], few studies have evaluated the cost-effectiveness of patient or provider-directed interventions for CRC screening in average risk patients, especially in the VA population [15-20]. The primary aim of this study is to assess the costs and cost-effectiveness of our mailed educational reminder on adherence with FOBT-type screening among the U.S. Veteran population.

## Methods

Data for this study were collected in 2007 and analyses were conducted in 2008-2009. Detailed descriptions of the design, methods, primary, and secondary outcomes of the randomized controlled trial on using a mailed educational reminder to increase FOBT card return rates have been published [12]. Here we provide a brief summary of the clinical trial and analytic methods below

### Clinical Trial

To test the effect of a mailed educational reminder on increasing FOBT card return rate for CRC screening, we conducted a double-blind, randomized controlled trial in a U.S. Veteran patient population. Patients meeting inclusion criteria (age ≥ 50, not up to date with CRC screening) were randomly assigned to receive either usual care or usual care with a mailed educational reminder (intervention), which was mailed 10 days after the patients received their FOBT cards from the laboratory. All patients were given 6 months to return the FOBT cards.

### Study Setting and Population

We evaluated patients from three VA primary care clinics in San Diego and Vista, California between June 1 and September 9, 2007. The study included asymptomatic men and women age 50 years or older who agreed with screening and received FOBT card kits with a postage paid return envelope. Under usual care, primary care physicians (PCP) entered a computerized order for FOBT for CRC screening, and then patients were instructed by their PCP to pick up FOBT cards from the laboratory and return them for analysis. Patients were excluded from this study if they were less than 50 years of age, were currently on an inpatient unit, were up-to-date with CRC screening, or refused to undergo any routine CRC screening. We did not exclude any patients over the age of 75 as recommended by the USPSTF in 2008, because our study was performed prior to the 2008 guidelines [6].

### Intervention

The mailed educational reminder consisted of an 8.5 × 11 paper folded in thirds, personalized, sealed, and sent to the subjects' home address 10 days after the patients were given their FOBT cards by the clinical laboratory (Additional file 1, **Appendix 1**). The mailed letter was one-sided, written at an eighth-grade reading level [21], and contained a reminder to return their FOBT cards on the top portion of the letter. The middle portion of the letter had several statements regarding the risk of developing CRC, who is affected by the disease, and the benefits of getting screened. On the bottom third of the letter was a quote from a United States Veteran colon cancer survivor, who emphasized the importance of colorectal cancer screening. The Institutional Review Board (IRB) at our institution granted exemption of an informed consent because of the minimal risk associated with the mailed reminder and a guarantee of at least usual care for all patients.

### Cost Analysis

An analysis of costs was conducted from the payer's perspective to help better represent the costs that a healthcare system may incur when offering interventions of this type. The payer's perspective will allow healthcare organizations to gauge approximately what it would cost for them to conduct a similar program in the future; assuming adjustments are made for inflation. Intervention costs were based on the actual personnel time and materials used in the mailed reminder and are detailed in the next section. All research related activities were excluded from the cost analysis.

### Costs of the Intervention

To calculate the costs of our intervention, we identified all inputs used for the intervention. The cost of our intervention included the cost of creating the letter content by a physician, editing and approving the content by management (Gastroenterology (GI) section chief), the time to generate and personalize the mailed reminders by an administrative assistant, overhead costs, and finally the cost of the mailed reminder itself including paper, envelopes, pen, printer ink, and postage (Table 1). Personnel cost estimates were derived from actual salary and benefit data, which was $40,000 per year for an administrative assistant, $150,000 per year for a physician, and $200,000 per year for a VA staff gastroenterologist. The labor costs were calculated by multiplying the time spent of performing the task (creating the letter content, editing and approving the content, generating, personalizing, and mailing the reminder) by the employee's wage per hour. We determined the costs of all materials used for the mailed reminder by multiplying the costs of each material by the number of patients receiving the mailed reminder. Overhead costs were estimated at 69% of the personnel costs required to deliver the intervention, which accounts for facilities costs, indirect support personnel, and other typical indirect costs associated with running an outpatient healthcare program [22]. This figure of 69% is based on data showing that only half of all healthcare reimbursement costs are related to direct provision of care and that roughly 69% of non-care costs are indirect costs [23,24]. This method has been used in the cost analyses of other similar trials [25]. Other costs, such as those associated with research and development (R&D) activity were excluded. First-copy costs, defined as costs incurred in establishing an intervention, are considered quasi-fixed costs independent of the number of units produced once production is started [26]. In general, first-copy costs are excluded when they involve situations where much of the intervention is already in existence and only modification is needed to adapt it for implementation [14]. This is assuming that the final product can be made available to other health care organizations through public access [14]. Furthermore, it is expected that managed care organizations or community clinics would not develop their own mailed reminder but would rely on public access or licensing arrangements.

**Table 1:** Cost of the Mailed Educational Reminder (Intervention)

Item Costs	Provider	Patients	Time (h)	Cost/h	Total $ Cost	Sensitivity Analysis (± 10%)	Sensitivity Analysis (± 25%)
**Startup Costs**							
Creating the letter content	Physician*		0.25	$72.12	$18	$64.80-79.20	$13.50-22.50
Management Costs (editing & approving the reminder)	Chief GI Physician**		0.25	$96.12	$24	$21.60-26.40	$18.00-30.00
**Total Startup Costs**					$42	$37.80-46.20	$31.50-52.50
**Maintenance Costs**							
Generating, personalizing & mailing the reminder Overhead (69% of personnel costs	Assistant***	387	0.05	$19.23	$372	$334.80-409.20	$279.00-465.00
personnel costs		387			$286	$257.40-314.60	$214.50-357.50
Envelopes ($0.10)		387			$39	$35.10-42.90	$29.25-48.75
Reminder ($0.10)		387			$39	$35.10-42.90	$29.25-48.75
Postage ($0.42)		387			$163	$146.70-179.30	$122.25-203.75
Printer ink ($0.05)		387			$19	$17.10-20.90	$14.25-23.75
Pens					$2	"tabcaption".80-2.20	"tabcaption".50-2.50
**Total Maintenance Costs**					**$920**	$828.00-1012.00	$690.00-1150.00
**Total Cost**					**$962**	$865.80-1058.20	$721.50-1202.50
**Total Cost/Patient**					**$2.49**	$2.24-2.74	"tabcaption".87-3.11

* Based on physician salary for a primary care physician at the VA: $150,000

** Based on VA gastroenterology physician salary: $200,000

*** Based on administrative assistant salary at the VA: $40,000

### Cost-effectiveness Analysis

The incremental cost-effectiveness ratio (ICER) was derived based on the difference in the costs assigned to the intervention and control groups divided by the difference in FOBT card return rates between the intervention and control groups. The result is a measure of efficiency, reported as dollars per additional patient screened. The lower the result (ICER), the more efficient is the intervention. Sensitivity analysis estimates were based on a 10% and 25% variation around the point estimates for the various cost inputs (i.e. labor costs, material costs, the effectiveness of the intervention) [27]. In addition, the ICER was similarly calculated for various subgroups including gender, race, age, current illicit drug use, current alcohol use, current tobacco use, psychiatric disorders, and history of prior FOBT completion for CRC screening. Lastly, we reviewed the literature and compiled a list of other ICER for patient-directed CRC screening promotion interventions.

## Results

### Study Participants

Among 846 patients who received FOBT cards for CRC screening during our 3-month enrollment period, 769 were determined eligible and were randomized to receive either a mailed educational reminder (n = 387) or usual care (n = 382). The baseline demographic and clinical characteristics were well matched in both the control and intervention groups [12]. The mean age of our study participants was 63.1 years (SD = 9.6). The majority of our study participants were male (96.3%), Caucasian (72.8%), and not married (56.6%); the largest minority group in the cohort was African-Americans (11.7%). Many members of the cohort were currently drinking alcohol (45.0%) and smoking tobacco (27.6%), but only a few were noted to be currently or recently using illicit drugs (6.5%). Major psychiatric disorders present in our cohort were anxiety disorders (11.3%) and mood disorders (28.9%).

### Effectiveness of the Intervention

**Figure 1:**
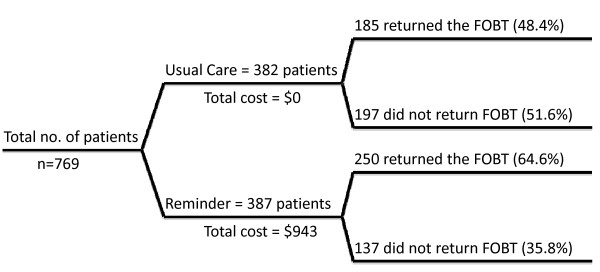
**Study Diagram of Intervention to Promote Colorectal Cancer Screening**. Flow diagram and results of randomized trial of intervention vs. usual care for colon cancer screening. 769 patients were enrolled and 382 were randomized to the control group, which received FOBT alone, and 387 patients were randomized to the intervention group, which was a mailed reminder 14 days after receiving their FOBT. The FOBT card return rate after 6 months was 64.6% in the intervention group compared to 48.4% in the usual care group (p < 0.001).

The proportion of patients who returned the FOBT cards was significantly higher in the mailed reminder group than those who did not receive a reminder. At 6 months after card distribution, the FOBT card return rate in the intervention and control arms were 64.6% (250/387) and 48.4% (185/382), respectively (P < 0.001) (Figure 1). The percent incremental effect was 16.2 percentage points and the absolute difference in FOBT card returned was 65. The timing of the return of FOBT kits was previously published, and the data indicated that 39-49% of patients returned kits by 30 days in the usual care and intervention control arms, respectively; and this increased to 47-62% by 90 days and to 48-65% by 180 days [13].

### Intervention Costs

The total cost of the intervention was $962, which is $2.49 per patient (Table 1). Our startup costs, which consisted of labor costs of creating the letter content by a physician and editing and approving the content by management (GI physician) came out to be $42. Our maintenance costs were $920, and consisted of labor costs from the administrative assistant who generated and personalized the mailed reminders, overhead costs (69% of personnel costs), and the cost of the mailed reminder itself including paper, envelopes, pen, printer ink, and postage. The time spent by a physician on researching and creating the content of the letter was about fifteen minutes. The time spent on editing and approving the content of the reminder was about fifteen minutes. Lastly, the time spent on generating, personalizing, and mailing the reminder was estimated to be around 3 minutes. Sensitivity estimates based on a 10% variation around the cost inputs revealed a total cost of the intervention ranging from $865.80 - $1058.20. Note that patient-related calls to administrative or nursing staff were not measured, but may have contributed to maintenance costs and the 10-25% variation estimates.

### Cost-effectiveness

The incremental cost-effectiveness ratio (ICER) for the mailed educational reminder (intervention) was $15 per additional person screened (Table 2). The ICER was obtained from the difference in costs per patient between the intervention and control groups ($2.49) divided by the difference in FOBT card return rates between the intervention and control groups (16.2%). Since costs included in this intervention may vary in different locations, we performed a sensitivity analysis for costs based on a 10% to 25% variation around the cost inputs (Table 1 and Table 3). Sensitivity estimates based on a 10% variation revealed that the estimated cost per additional patient screened for CRC ranged from $13.50 to $16.50. Sensitivity estimates based on a 25% variation revealed that the estimated cost per additional patient screened for CRC ranged from $11.25 to $18.75. For comparison, ICERs for other published patient directed interventions for CRC screening are listed in Table 4. In order to determine how differences in screening compliance in different patient subgroups affected the cost-effectiveness, we performed a subgroup analysis. As depicted in Table 3, the intervention had a similar ICER, ranging from $5.65 to $27.07 per additional person screened across various subgroups of patients. Interestingly, minorities such as African-Americans and Asians appear to have the lowest ICER in our subgroup analyses for reasons that remain unclear. Further studies are needed to help understand and confirm this finding.

**Table 2:** Incremental Cost-effectiveness (Intervention cost per additional individual screened)

Intervention	Cost	Incremental cost	Effect % Screened	Incremental effect, %	Incremental cost-effectiveness
Usual Care (control)	0	-	48.4	-	-
Mailed Reminder	$2.49	$2.49	64.6	16.2	$15

**Table 3:** ICER of the Mailed Reminder Intervention by Subgroup

Subgroups	Usual care % FOBT card return rate	Mailed reminder % FOBT card return rate	% Effect	Marginal Cost	ICER	Sensitivity Analysis **(**±**10%)**	Sensitivity Analysis **(**±**25%)**
All patients, n = 769	48.4%	64.6%	16.2%	$2.49	$15.00	$13.50-16.50	$11.25-18.75
Race							
White, n = 560	52.2%	61.6%	9.4%	$2.49	$26.49	$23.84-29.14	$19.87-33.11
Black, n = 90	35.0%	77.6%	42.6%	$2.49	$5.85	$5.26-6.44	$4.39-7.31
Hispanic, n = 58	40.0%	51.9%	11.9%	$2.49	$20.92	$18.83-23.01	$15.69-26.15
Other (Asians, etc), n = 61	41.1%	85.2%	44.1%	$2.49	$5.65	$5.08-6.22	$4.24-7.06
Males, n = 741	47.4%	64.4%	17.0%	$2.49	$14.65	$13.18-16.12	$10.99-18.31
Females, n = 28	60.0%	69.2%	9.2%	$2.49	$27.07	$24.36-29.78	$20.30-33.84
Age							
50 - 59 years, n = 336	40.5%	60.1%	19.6%	$2.49	$12.70	$11.43-13.97	$9.52-15.88
60 - 69 years, n = 250	50.0%	66.4%	16.4%	$2.49	$15.18	$13.60-16.70	$11.38-18.98
>70 years, n = 183	59.8%	70.9%	11.1%	$2.49	$22.43	$20.19-24.67	$16.82-28.04
Current illicit drug use, n = 50	20.8%	39.1%	18.3%	$2.49	$13.61	$12.25-14.97	$10.21-17.01
Current alcohol use, n = 346	50.3%	62.1%	16.7%	$2.49	$14.91	$13.42-16.40	$11.18-18.64
Current tobacco use, n = 212	35.4%	60.3%	11.8%	$2.49	$21.10	$18.99-23.21	$15.82-26.38
Psychiatric disease							
Anxiety disorder, n = 87	57.1%	66.7%	9.6%	$2.49	$25.94	$23.35-28.53	$19.45-32.43
Mood disorder, n = 222	40.4%	58.9%	18.5%	$2.49	$13.46	$12.11-14.81	$10.09-16.83
Psychotic disorder, n = 22	54.5%	36.4%	-18.1%	$2.49	-	-	-
No. of prior FOBT completed							
None, n = 384	39.6%	49.5%	9.9%	$2.49	$25.15	$22.63-27.67	$18.86-31.44
1, n = 187	49.4%	69.8%	23.4%	$2.49	$10.64	$9.58-11.70	$7.98-13.30
2, n = 90	64.3%	87.2%	22.9%	$2.49	$10.87	$9.78-11.96	$8.15-13.59
3, n = 108	70.2%	86.2%	16.0%	$2.49	$15.56	$14.00-17.12	$11.67-19.45

**Table 4:** ICERs for other Patient-Directed Interventions to Improve Colorectal Cancer Screening

Study	Patients	Target Population	Target CRC screening test	Cost of Intervention	Intervention	Baseline Screening Rate	Change in Effectiveness	ICER
Lee et al. 2009	775	Veterans aged ≥ 50 years who were average risk for CRC	FOBT	Mailed Reminder	$2.49	48.4%	16.2%	$15
Lewis et al. 2008	237	Patients in a University-based practice age ≥50 years who were average risk for CRC	FOBT, FS, colonoscopy	Mailed package that included a letter from their PCP, a CRC screening decision aid, and instructions for obtaining each screening test without an office visit	$11	4%	11%	$94
Lairson et al. 2007	1546	Patients in a University-based practice ages 50-74 years who were average risk for CRC	FOBT, FS, colonoscopy barium enema	Patients randomized into 4 groups: control, standard group (SI) consisting of mailed informational brochure, invitation letter, FOBT cards, and reminder letter; tailored intervention (TI) consisting of standard intervention plus motivational messages based on patient-specific survey data; tailored interventions (TIP) consisting of tailored intervention plus reminder phone call	$42 for SI $150 for TI $200 for TIP	33%	13% for SI TI and TIP did not yield much significant change in effective ness	$319 for SI $5842 for TIP
Shankaran et al. 2006	781	Patients in a University-based practice ages ≥50 years who received referrals for screening colonoscopy	Colonoscopy	Mailed informational Brochure/Reminder	$5	59%	12%	$43
Sequist et al. 2010	21,860	Patients aged 50-80 years across 11 health centers who were average risk for CRC	FOBT, FS, colonoscopy	Patient mailing with FOBT kit, telephone line to schedule colonoscopy, and mailed reminder 6 months later	$5.48	38%	6%	$94

FOBT: Fecal occult blood test, CRC: Colorectal cancer, FS: Flexible sigmoidoscopy

## Discussion

Our economic analysis demonstrates that a low-intensity patient-directed intervention is inexpensive, effective, and cost-effective. A simple mailed educational reminder, mailed 10 days after patients picked up their FOBT cards from the laboratory, significantly increased FOBT card return rate by 16.2% compared to the control group (64.4% vs. 48.4%, P < 0.001) [12]. The total cost of our intervention was $962, which came out to be $2.49 per individual in our study population. More importantly, the incremental cost of our intervention was $15 per additional individual screened for CRC, which is markedly lower compared to other patient-directed interventions for CRC screening (Table 4). In the past 15 years, several studies have demonstrated the effectiveness of tailored interventions in increasing CRC screening compliance, but few have examined these interventions from a cost perspective [8,10,15,28,29]. To date, our study is the first study that estimates the costs and cost-effectiveness of a patient-directed intervention (mailed educational reminder) on CRC screening among U.S. Veterans at average risk of developing CRC.

Prior studies on cost-effectiveness of patient-directed interventions for CRC screening have shown mixed results (Table 4), with costs ranging from $43 to $5842 per additional individual screened for CRC [17,18,20,27]. In one study, Lairson and colleagues randomized their patients who received FOBT and flexible sigmoidoscopy (FS) referrals to either two rounds of mailed reminders, two rounds of tailored mailed reminders, or two rounds of reminder telephone calls along with the tailored mailed reminder [18]. Their results showed that increasingly intensive patient-directed interventions resulted in higher ICERs, with the most unfavorable ICER of $5842 coming from the addition of a reminder telephone call to a tailored mailed reminder [18]. The ICER of two rounds of mailed reminders was $319 per additional patient screened [18]. There are several reasons for the higher ICER compared to our study including the number of mailed reminders (two reminders), which increased labor, supply, and postage costs, the lower incremental effect (13.2% compared to our 16.2%) from their intervention, and the costs of mailing the FOBT cards to every study participant [18].

In a study at a university-based ambulatory care center, Lewis et al. implemented a multi-modal intervention, which included a letter from their PCP, a colon cancer screening decision aid, and instructions for obtaining each screening test without an office visit so that patients could access screening tests directly [17]. Although effective in increasing CRC screening rates by 11%, the ICER for that intervention was $94 per extra person screened for CRC [17]. Despite the significant increase in CRC screening rates, there were several limitations to the study including a modest sample size (n = 237), non-randomized study design, and the difficulty to determine the relative degree to which compliance was influenced by the reminder itself, the colon cancer screening decision aid, or the removal of system barriers by eliminating office visits. It is also possible that these additional materials have benefits beyond increased screening rates.

Recently, Sequist et al. demonstrated that mailing a tailored letter along with an FOBT kit improved CRC screening rates by 6% [20]. In this study, 21,860 patients from 11 health centers in Eastern Massachusetts were randomized to receive a mailing that included a tailored letter, an educational brochure, a dedicated telephone number to schedule a flexible sigmoidoscopy or colonoscopy, and an FOBT kit. Participants that were still overdue for CRC screening 6 months after initial enrollment of the study received a mailed reminder. The cost of the intervention was $5.48 per patient and the ICER was $94 per additional patient screened. Although the study showed promising results, the intervention could only be applied in a large integrated medical group with a well-established electronic health record. In addition, the improvement in screening rates was about one half of what we observed in our study, and as a result, the ICER was much higher than what we report. Similar to our study, although not related to FOBT adherence, Shankaran et al. were able to show that a 1-page, 2-sided mailed reminder significantly increased colonoscopy appointment adherence by 12% and had an ICER of $43 per additional person screened for CRC [27]. However, the cost inputs for Shankaran's study were not reported clearly, particularly the initial data costs, potentially making the intervention difficult to replicate [27].

Personnel cost, particularly using our administrative assistant to generate and mail the reminder, were the most significant cost to our intervention. With rising costs of health care, one should consider if available, information technology systems that could generate mailed reminders from electronic medical records. This change would significantly decrease the ICER by reducing the number of minutes spent by the administrative assistant. Given the ease of electronic data acquisition within the VA healthcare system, we view this to be the next logical step for intervention implementation.

In recent years, several health maintenance organizations (HMO), clinics, and primary care physicians have started using fecal immunochemical tests (FIT) to improve CRC screening rates. However, studies have shown that compliance rates with FIT are either slightly better or similar compared to the guaiac-based tests [30-32]. Knowing that FIT kits are much more expensive compared to the guaiac-based tests, healthcare organizations will likely face higher costs associated with patients not returning the FIT kits compared to the guaiac-based tests. Although there have not been any studies evaluating the effectiveness of a mailed reminder on FIT kit return rates, it is likely that our intervention could similarly improve FIT kit return rates, thus lowering the costs associated with non-compliance.

There are several limitations to our study. First, our study was a cost-effectiveness analysis with an intermediate outcome (cost per additional person screened for CRC). We did not use the standard metric for cost-effectiveness, which is the cost per year of life saved. The goal of our study was to increase the utilization of FOBT, a CRC screening method that has already been shown to be cost-effective. We understand that cost-effectiveness ratios do not provide a definitive answer, but it can assist the decision maker in choosing the most appropriate intervention given the cost, effectiveness, and resources available to implement the program. Secondly, our sample was comprised of U.S. Veteran patients from San Diego, California, which limits the generalizability of our findings. More studies are needed at other VA medical centers, community based medical centers, and non-VA settings to help establish the intervention's generalizibility. Third, our study was based on an analysis from the payer perspective, and costs incurred by individual patients were not included in the analysis. Fortunately, FOBT costs to the patient are negligible in that the patient can perform the test quickly in his or her home with minimal discomfort or complications. Lastly, we did not exclude any patients over the age of 75 as recommended by the USPSTF in 2008 [6], because our study was performed prior to these recommendations.

## Conclusion

In conclusion, a simple mailed educational reminder increased FOBT card return rate at a cost many health care systems can afford. The costs and ICER associated with this patient-directed intervention was markedly lower than most analogous CRC screening interventions and therefore could be recommended for implementation at this time in practices with similar organizational and patient characteristics. Cost analyses on promoting CRC screening interventions should be emphasized in increasing CRC screening rates in the U.S.

## Competing interests

The authors declare that they have no competing interests.

## Authors' contributions

JKL designed the study, analyzed the data, wrote and edited the manuscript. EJG designed the study, analyzed the data. TGG designed the study and edited the final manuscript. SBH designed the study, analyzed the data, wrote and edited the manuscript All authors have approved the submitted final draft.

## Pre-publication history

The pre-publication history for this paper can be accessed here:

http://www.biomedcentral.com/1471-230X/11/93/prepub

## Supplementary Material

Additional file 1 **Appendix Mailed Educational Reminder**. The mailed educational reminder used in the intervention. Click here for file
