# Supplementary material for: Cost-effectiveness of a mailed educational reminder to increase colorectal cancer screening
Source: BMC Gastroenterol. 2011 Aug 25;11:93. doi: 10.1186/1471-230X-11-93 (PMC3179931; doi:10.1186/1471-230X-11-93)
Supplement: Additional file 1 — Appendix Mailed Educational Reminder. The mailed educational reminder used in the intervention. [file 1471-230X-11-93-S1.DOC]

Reminder!

Please return the stool cards that you were given to test for colon cancer. Thank you.

If you have any questions about how to do the tests, please call the VA lab 24 hrs/7 days a week at (858) 642-3426 or 1-800-331-VETS (8357) ext. 3426


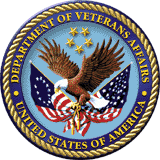


**Why is this important?**

- Colon cancer is the 2^nd^ leading cause of cancer deaths in the United States.
- 1 in 20 adults will develop colon cancer in their lifetime.
- Everyone is at risk for colon cancer.
- Colon cancer occurs most often in people age 50 and older. Your risk increases with age.
- You could have this “silent disease” and not have any symptoms.
- If you catch colon cancer early, it is 90% curable. If you catch it late, it is only 8% curable.


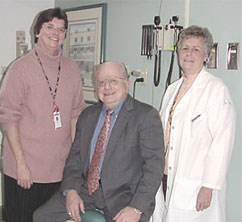


“Don’t wait for symptoms. The few minutes you go through completing the test can save you months, years, your life!”

U.S. Veteran Edward Ketterer from New York, colon cancer survivor
